# Hysteroscopy for Retained Products of Conception in Japan: A Survey of Clinical Practice and Educational Needs

**DOI:** 10.1111/jog.70564

**Published:** 2026-11-08

**Authors:** Keita Yatsuki, Kazunori Nagasaka, Kensuke Sakai, Akiko Takashima, Kenbun Sone, Akemi Koshiba, Osamu Hiraike, Juichiro Saito, Wataru Yamagami

**Affiliations:** ^1^ Department of Obstetrics and Gynecology Teikyo University School of Medicine Tokyo Japan; ^2^ Future Planning Committee, Japan Society for Hysteroscopy Tokyo Japan; ^3^ Department of Obstetrics and Gynecology Keio University School of Medicine Tokyo Japan; ^4^ Department of Obstetrics and Gynecology Toho University Sakura Medical Center Chiba Japan; ^5^ Department of Obstetrics and Gynecology Toho University Omori Medical Center Tokyo Japan; ^6^ Department of Obstetrics and Gynecology Kyoto City Hospital Kyoto Japan; ^7^ Department of Obstetrics and Gynecology Nippon Medical School Hospital Tokyo Japan; ^8^ Department of Obstetrics and Gynecology, Juntendo Tokyo Koto Geriatric Medical Center Juntendo University School of Medicine Tokyo Japan

**Keywords:** continuing, education, hysteroscopy, medical, obstetricians, retained products of conception, surveys and questionnaires

## Abstract

**Aim:**

To characterize the use of hysteroscopy for the management of retained products of conception (RPOC) and identify perceived barriers and educational needs among members of the Japan Society of Perinatal and Neonatal Medicine.

**Methods:**

An anonymous online survey was conducted among members of the Japan Society of Perinatal and Neonatal Medicine from October 16 to November 15, 2024. Responses were summarized descriptively using questionnaire‐branch‐specific denominators.

**Results:**

Seventy‐seven perinatal clinicians responded, of whom 76 reported having diagnosed RPOC or suspected disease after delivery. Among these 76 respondents, 20 (26.3%) and 22 (28.9%) reported using diagnostic and operative hysteroscopy, respectively. Sixteen of the 20 diagnostic users (80.0%) reported obtaining useful information, and none reported complications during diagnostic hysteroscopy. Two operative users reported a complication or residual tissue, and one did not answer the complication question; these responses could not be used to estimate patient‐level complication rates. Among 56 respondents who reported not using hysteroscopy to diagnose RPOC, 20 (35.7%) expressed interest in using it. The most frequently reported barriers were equipment and environmental limitations (26/56, 46.4%) and safety concerns (24/56, 42.9%). Overall, 56 of the 77 respondents (72.7%) expressed interest in hands‐on seminars or training courses.

**Conclusions:**

Diagnostic and operative hysteroscopy for RPOC were each used by fewer than one‐third of respondents who answered the relevant management questions, whereas interest in training was common. These findings identify perceived barriers and educational needs that may inform hysteroscopy training and referral arrangements for RPOC care.

## 1 Introduction

Retained products of conception (RPOC) are an important consideration in patients presenting with persistent or recurrent bleeding after pregnancy. Diagnosis can be difficult because an intracavitary mass or abnormal vascularity does not invariably establish the presence of retained placental tissue. While ultrasonography, including color Doppler assessment, can provide information about both intracavitary findings and vascularity, an absence of Doppler flow does not exclude RPOC [1]. Therefore, decisions regarding further investigation and treatment require consideration of both imaging findings and clinical presentation.

Hysteroscopy allows direct inspection of the uterine cavity and targeted removal of the retained tissue. Observational evidence suggests that lesion appearance and vascularity may help clinicians anticipate required instruments and hemostatic measures [2]. However, the potential benefits of hysteroscopy depend on the clinical setting and the treatment used as a comparator. A randomized trial comparing hysteroscopic morcellation with ultrasound‐guided vacuum aspiration found more complete removal with morcellation but no significant difference in intrauterine adhesions [3]. Another randomized trial involving patients with incomplete spontaneous abortion did not demonstrate improved subsequent pregnancy outcomes with hysteroscopy [4]. These findings support careful consideration of hysteroscopy alongside other management options, based on the clinical context and treatment goals.

Japanese endoscopic surgery registry data describe the broader practice of hysteroscopic surgery [5], but do not establish how frequently physicians involved in perinatal care use hysteroscopy for RPOC, what barriers they encounter, or their educational needs. Understanding these aspects of clinical practice is important because an obstetrician assessing suspected RPOC may need to coordinate further treatment with a gynecologic endoscopist or interventional radiologist.

We surveyed members of the Japan Society of Perinatal and Neonatal Medicine regarding their reported use of diagnostic and operative hysteroscopy for RPOC. We also asked about other management approaches, reasons for nonuse, and interest in training. The aim was to characterize current practices, perceived barriers, and educational needs and to consider how the findings might inform training and referral arrangements.

## 2 Methods

### 2.1 Survey Design and Participants

The survey was planned and conducted by the Future Planning Committee of the Japan Society for Hysteroscopy. An anonymous, cross‐sectional online survey was conducted from October 16 to November 15, 2024. A notice on the Japan Society of Perinatal and Neonatal Medicine website invited members to complete a questionnaire administered using Google Forms. Because the number of eligible members who viewed the notice or accessed the questionnaire was unavailable, the response rate could not be calculated. The report was prepared with reference to recommendations for internet surveys and observational studies [6, 7]. The questionnaire was not pilot‐tested, and no incentives were offered.

### 2.2 Questionnaire and Response Branches

The 28 questions addressed professional experience and credentials, workplace characteristics, experience diagnosing RPOC after delivery, diagnostic methods and management policies, hysteroscopy use, perceived benefits and complications, reasons for nonuse, possible indications, and educational needs. Some questions allowed multiple responses, and workplace categories were not mutually exclusive.

Respondents who reported diagnosing or suspecting RPOC after delivery answered subsequent management questions. Those who did and did not use diagnostic hysteroscopy received different follow‐up questions; operative hysteroscopy was addressed in a separate branch. The diagnostic and operative hysteroscopy groups were not mutually exclusive. Twelve respondents reported both diagnostic and operative hysteroscopy use. The initial question concerned disease after delivery, whereas later questions used the broader term “RPOC” without distinguishing pregnancy outcomes.

### 2.3 Analysis

The analysis was descriptive. We summarized respondent‐level responses as numbers and percentages using the relevant questionnaire branch as the denominator, rounded to one decimal place. The full sample comprised 77 respondents; the management branch, 76; the diagnostic‐use and nonuse branches, 20 and 56, respectively; and the operative‐use and nonuse branches, 22 and 53, respectively, with one missing response regarding operative use. Unanswered responses were retained as separate categories. Percentages could sum to more than 100% for questions allowing multiple selections. Free‐text responses were categorized according to their content and incorporated into the corresponding response categories where applicable. Each respondent was counted only once per category, even when the same concept was expressed in both a selected option and free text. No missing values were imputed, and no subgroup comparisons, association tests, or multivariate analyses were performed. Complication reports reflected respondents' experiences across an unspecified number of procedures and could not establish patient‐level complication rates. Additional treatments were summarized as respondent counts among the 22 operative users. Treatment timing, indications, and combinations used in individual patients could not be determined.

### 2.4 Ethical Considerations

The anonymous survey concerned physicians' reported practices and opinions and was conducted with permission from the Japan Society of Perinatal and Neonatal Medicine. This report used survey responses and excluded individual patient‐case data and illustrative patient cases presented on conference slides. The invitation explained that findings would be disseminated in a non‐identifiable form at scientific meetings and in journals. It also stated that separate ethics reviews at respondents' institutions were unnecessary because individual patient‐case data were not collected.

### 2.5 Use of Artificial Intelligence

OpenAI (GPT‐6 astra) was used to assist with the English‐language editing of the manuscript. The authors reviewed and verified all AI‐assisted text and translations, made revisions as necessary, and take full responsibility for the accuracy and integrity of the final manuscript.

## 3 Results

### 3.1 Respondent and Workplace Characteristics

All 77 respondents reported specialist certification in obstetrics and gynecology. Forty‐eight (62.3%) had at least 20 years of experience, 55 (71.4%) had perinatal specialist certification, and 18 (23.4%) held certification in gynecologic endoscopic surgery. Fifty‐one respondents (66.2%) selected perinatal medical centers as their workplace category, 24 (31.2%) selected university hospitals, 17 (22.1%) selected community hospitals, and nine (11.7%) selected clinics (Table 1). Workplace categories were not mutually exclusive.

**TABLE 1:** Respondent and workplace characteristics.

Characteristic or response	*n*	%
Years of obstetric and gynecologic experience		
< 5	0	0.0
5–9	4	5.2
10–14	12	15.6
15–19	13	16.9
≥ 20	48	62.3
Professional credentials		
Obstetrics and gynecology specialist	77	100.0
Perinatal specialist	55	71.4
Certification in gynecologic endoscopic surgery	18	23.4
Ultrasound specialist	14	18.2
Workplace category		
Perinatal medical center	51	66.2
University hospital	24	31.2
Community hospital	17	22.1
Clinic	9	11.7
Full‐time anesthesiology coverage		
Available	68	88.3
Unavailable	9	11.7
Interventional radiology availability		
Always available	37	48.1
Available with restrictions	21	27.3
Unavailable	18	23.4
No response	1	1.3
Annual delivery volume		
< 500	33	42.9
500–999	35	45.5
≥ 1000	9	11.7

*Note: N* = 77 respondents. Values describe physicians and their reported workplaces, not unique institutions. Multiple selections were permitted; percentages may exceed 100% when summed. All percentages used 77 as the denominator, including the unanswered interventional radiology items.

Full‐time anesthesiologist coverage was reported by 68 respondents (88.3%). Interventional radiology was always available at the workplaces of 37 respondents (48.1%), available with restrictions for 21 (27.3%), and unavailable for 18 (23.4%); one response was missing. The most common annual delivery volume was 500–999 deliveries, reported by 35 respondents (45.5%).

### 3.2 Experience With Diagnosis and Management

Seventy‐six respondents (98.7%) reported diagnosing or suspecting RPOC after delivery. Of these, 40 (52.6%) reported fewer than 10 diagnoses during the preceding 5 years, 32 (42.1%) reported 10–49, three (3.9%) reported 50–99, and one (1.3%) reported at least 100. Bleeding was reported by 73 respondents (96.1%), infection by eight (10.5%). These figures describe clinicians' experiences, not patient‐level symptom frequencies.

All 76 respondents in the management branch reported using ultrasonography: 33 (43.4%), 28 (36.8%), and eight (10.5%) reported using magnetic resonance imaging, computed tomography, and angiography, respectively. Management was guided by symptoms for 61 respondents (80.3%) and imaging findings for 55 (72.4%). Thirty‐three respondents (43.4%) selected initial expectant management, including medical treatment. No respondents selected immediate intrauterine aspiration or curettage as a basic management policy. Ten respondents (13.2%) reported difficulty in selecting a management approach because they lacked an established policy (Table 2).

**TABLE 2:** Reported experience with RPOC diagnosis and basic management.

Characteristic or response	*n*	%
RPOC diagnoses in the preceding 5 years		
< 10	40	52.6
10–49	32	42.1
50–99	3	3.9
≥ 100	1	1.3
Clinical presentations encountered		
Bleeding	73	96.1
Infection	8	10.5
Diagnostic modalities used		
Ultrasonography	76	100.0
Computed tomography	28	36.8
Magnetic resonance imaging	33	43.4
Angiography	8	10.5
Basic management policy		
Guided by imaging findings	55	72.4
Guided by symptoms	61	80.3
Initial expectant management including medication	33	43.4
Immediate aspiration or curettage	0	0.0
Consider office hysteroscopic removal or transcervical resection	6	7.9
Adapt to patient preferences	5	6.6
No established policy and difficulty deciding	10	13.2
Referral to another institution	2	2.6

*Note: N* = 76 respondents with diagnosed or suspected retained products of conception (RPOC) after delivery. Multiple selections were permitted. Counts represent respondents who selected a category rather than the number of patients, episodes, or procedures.

### 3.3 Diagnostic Hysteroscopy

Twenty of the 76 respondents (26.3%) reported using hysteroscopy to diagnose RPOC: 13 used a flexible hysteroscope, and seven a rigid hysteroscope. Sixteen (80.0%) reported obtaining useful information, whereas four did not. None reported complications during diagnostic hysteroscopy (Table 3). The questionnaire did not collect procedural totals or systematic follow‐up data.

**TABLE 3:** Reported diagnostic and operative hysteroscopy experience.

Characteristic or response	*n*	%
Diagnostic hysteroscope mainly used (*N* = 76)		
Flexible	13	17.1
Rigid	7	9.2
No diagnostic use	56	73.7
Useful information obtained (*N* = 20)		
Yes	16	80.0
No	4	20.0
Diagnostic complication experienced (*N* = 20)		
Yes	0	0.0
No	20	100.0
Operative hysteroscopy reported (*N* = 76)		
Yes	22	28.9
No	53	69.7
No response	1	1.3
Operative method (*N* = 22)^a^		
Transcervical resection	22	100.0
Office surgery	1	4.5
Surgical complication or adverse outcome (*N* = 22)^b^		
Reported	2	9.1
Not reported	19	86.4
No response	1	4.5
Reported adverse‐outcome categories (two positive respondents)^b^		
Infection	0	
Bleeding	1	
Perforation	0	
Residual tissue	1	
Additional treatment reported (*N* = 22)		
Yes	11	50.0
No	11	50.0
Additional‐treatment categories (22 surgical users)^a^, ^c^		
Endometrial curettage	4	
Uterine artery embolization	8	
Gonadotropin‐releasing hormone antagonist	1	
Hormonal preparations including estrogen–progestin	2	
Transfusion	4	
Hysterectomy	0	

*Note:* Denominators are specified for each item.

Abbreviation: RPOC, retained products of conception.

^a^ More than one selection was possible.

^b^ Respondent‐level experiences rather than patient‐level event rates. Residual tissue indicates incomplete clearance and is reported separately from bleeding.

^c^ Counts indicate the number of respondents reporting each additional treatment. Eleven of the 22 operative users reported additional treatment, and multiple selections were permitted. Treatment timing, indications, and combinations used in individual patients could not be determined. Planned or staged interventions could therefore not be distinguished from interventions undertaken to manage complications. Percentages are not shown.

Of the 56 respondents who did not use hysteroscopy to diagnose RPOC, 20 (35.7%) expressed interest in using it, whereas 36 (64.3%) did not (Figure 1). The reported barriers included equipment or environmental concerns (26/56, 46.4%), complications or safety concerns (24/56, 42.9%), uncertainty about usefulness (17/56, 30.4%), and technical concerns (10/56, 17.9%). Four respondents considered hysteroscopy unnecessary, and one cited inconvenience (Figure 2A).

**FIGURE 1:**
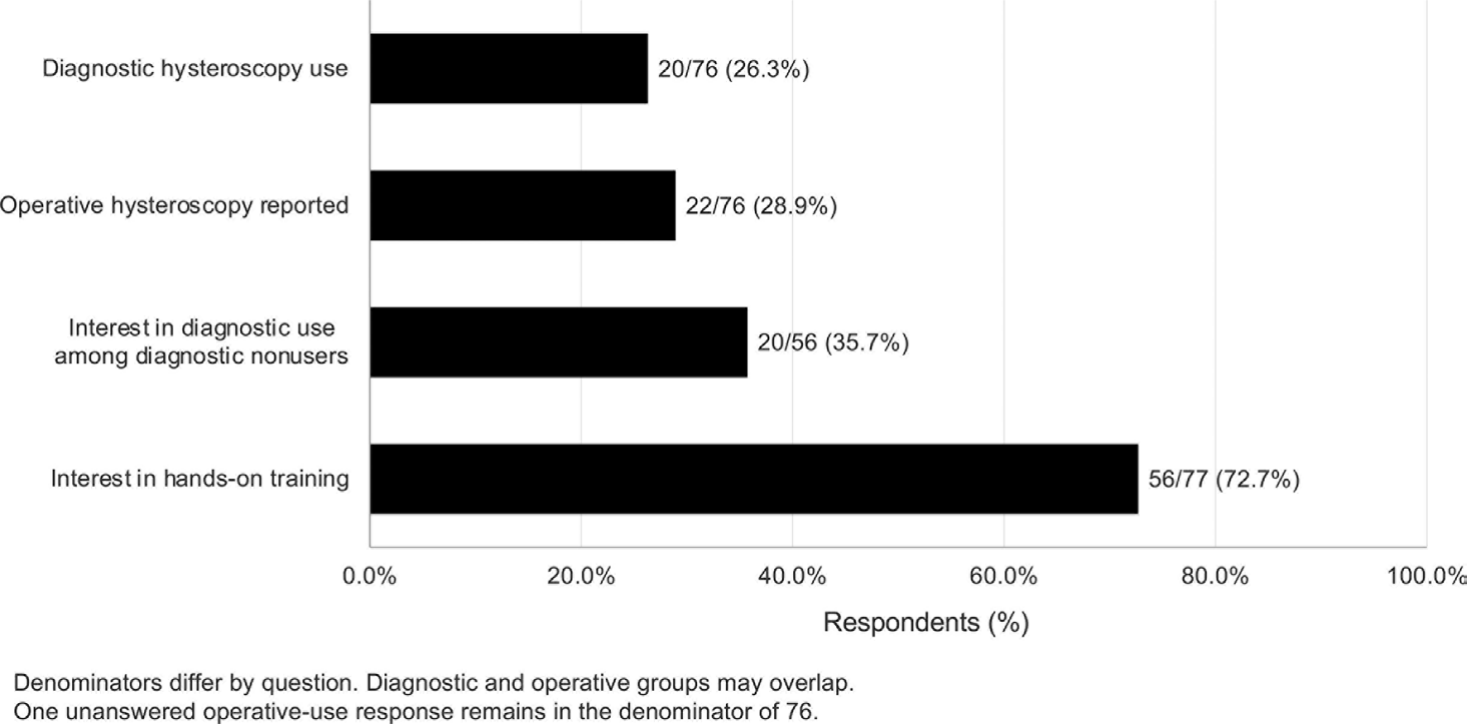
Reported hysteroscopy use and interest in implementation or education. Bars show the percentage selecting each response, with the count and corresponding denominator displayed. Diagnostic and operative use are based on 76 respondents with experience diagnosing retained products of conception (RPOC) or suspected RPOC after delivery. Interest in diagnostic use is based on 56 diagnostic nonusers. Interest in training is based on all 77 respondents. One of 76 respondents did not answer the operative‐use question and remains in that denominator. The four rows describe different questions and partly overlapping populations; no between‐row statistical comparison was performed.

**FIGURE 2:**
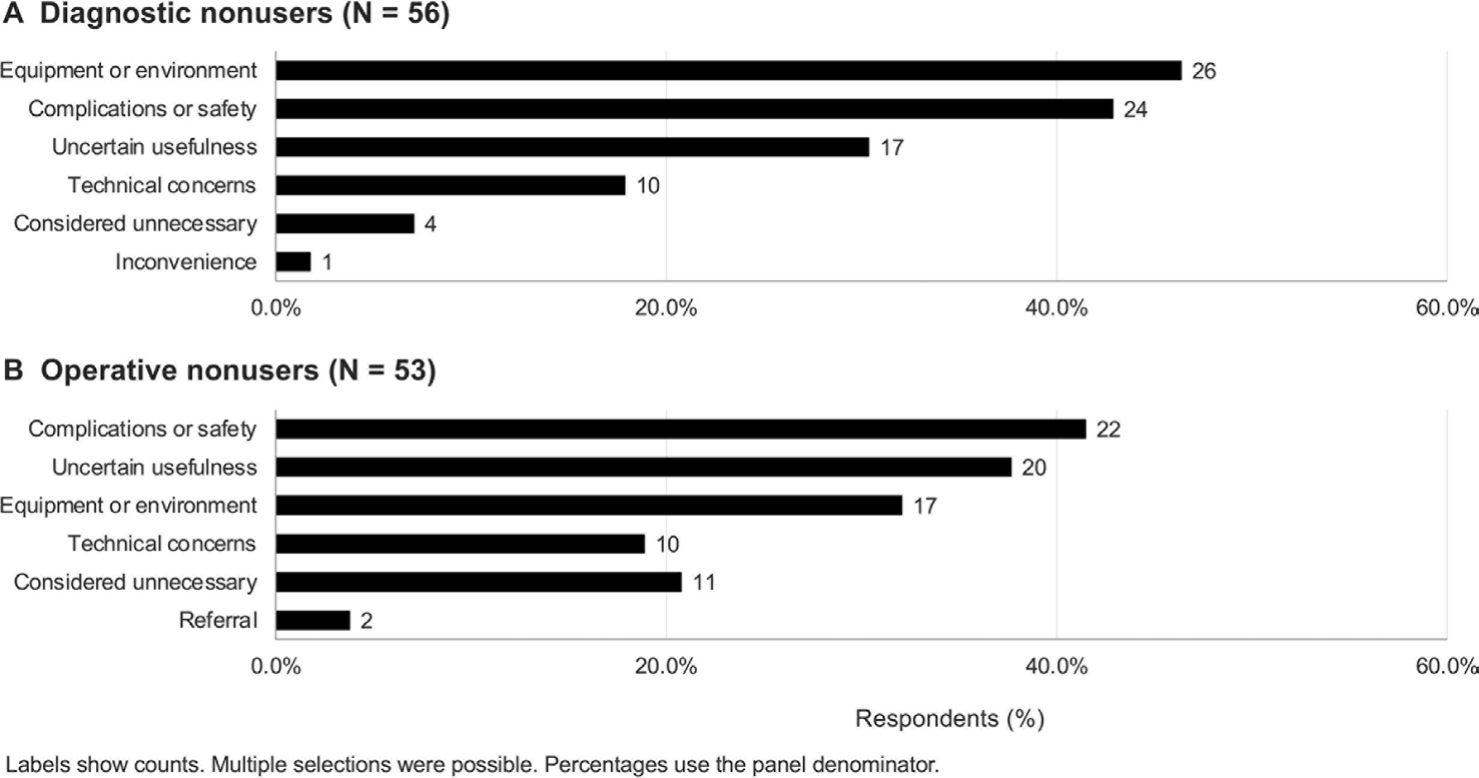
Reported reasons for not using hysteroscopy for retained products of conception. (A) Fifty‐six diagnostic nonusers. (B) Fifty‐three operative nonusers. Bars show percentages of the corresponding branch, with counts displayed beside each bar. More than one category could be reported, and percentages may exceed 100% when summed. Categories reflect the questionnaire response options and categorized free‐text responses, translated into English. The populations may overlap; the figure does not represent a paired comparison.

### 3.4 Operative Hysteroscopy and Additional Treatment

Twenty‐two of the 76 respondents (28.9%) reported performing hysteroscopic surgery after RPOC diagnosis, 53 (69.7%) reported not performing it, and one did not answer. All 22 operative users selected transcervical resection, and one also reported office hysteroscopic surgery (1/22, 4.5%). Two reported complications or adverse outcomes, 19 reported none, and one did not answer. Bleeding and residual tissue were each reported once; neither infection nor perforation was reported. These responses could not establish complication rates per procedure or overall surgical success (Table 3).

Eleven of the 22 operative users (50.0%) reported requiring additional treatment, including uterine artery embolization (*n* = 8), endometrial curettage (*n* = 4), transfusion (*n* = 4), hormonal preparations such as estrogen–progestin combinations (*n* = 2), and gonadotropin‐releasing hormone antagonist (*n* = 1). No respondent selected hysterectomy. Multiple selections were possible. The survey did not establish whether additional treatments were planned, staged, or undertaken to manage acute complications.

Among the 53 respondents who did not perform operative hysteroscopy, 21 (39.6%) cited safety concerns, 19 (35.8%) uncertainty about usefulness, 17 (32.1%) equipment or environmental concerns, and 10 (18.9%) technical concerns. Eleven respondents (20.8%) considered surgery unnecessary, and two (3.8%) reported referral (Figure 2B). Other reported management approaches included uterine artery embolization (27/53, 50.9%), observation without treatment (16/53, 30.2%), endometrial curettage (8/53, 15.1%), and hormonal preparations (10/53, 18.9%). Additional survey findings are summarized in Table S1, and the complete de‐identified respondent‐level responses are provided in Data S1.

### 3.5 Perceived Indications and Educational Needs

Among all 77 respondents, the desire for another pregnancy was the most frequently selected indication for office (30/77, 39.0%) and operative (35/77, 45.5%) hysteroscopy. Other frequently selected office indications included asymptomatic disease (23/77, 29.9%) and unchanged imaging findings (18/77, 23.4%). For surgery, unchanged imaging findings (29/77, 37.7%) and symptoms such as bleeding (26/77, 33.8%) were frequently selected. Seventeen respondents (22.1%) were unsure about office indications, and 16 (20.8%) were unsure about surgical indications (Table S1). Fifty‐six respondents (72.7%) expressed interest in hands‐on seminars or training courses on hysteroscopy for RPOC, whereas 21 (27.3%) did not (Figure 1).

## 4 Discussion

This survey identified infrequent reported use of hysteroscopy for RPOC among a self‐selected group of perinatal clinicians with substantial obstetric experience. Approximately one‐quarter of respondents who had diagnosed RPOC had used diagnostic hysteroscopy, and fewer than one‐third reported performing hysteroscopic surgery. Most respondents who used diagnostic hysteroscopy considered the information useful, and nearly three‐quarters of all respondents expressed interest in training. Equipment and environmental concerns, safety concerns, and uncertainty about hysteroscopy's usefulness were commonly reported barriers. These findings highlight opportunities for educational and service improvements.

Substantial clinical experience did not necessarily correspond to frequent RPOC exposure. More than half of the respondents in the management branch reported fewer than 10 diagnoses over 5 years. Infrequent exposure may make it difficult to maintain procedural confidence. The relationship between RPOC case exposure and hysteroscopy use was not examined in this descriptive analysis.

All 22 respondents who reported performing operative hysteroscopy selected transcervical resection, whereas Wagenaar et al. [3] evaluated hysteroscopic mechanical morcellation in a randomized trial. Among 133 patients with ultrasound‐detected RPOC measuring 1–4 cm, hysteroscopic morcellation achieved complete removal more often than ultrasound‐guided vacuum aspiration (95.2% vs. 82.5%). Intrauterine adhesions were detected in 14.3% and 20.6% of patients, respectively, without a statistically significant difference [3]. Differences in intervention schedules between groups also limit the attribution of the outcomes solely to the instrument used.

The larger HY‐PER randomized trial found no improvement in subsequent pregnancies lasting at least 22 weeks after hysteroscopy versus vacuum aspiration among patients with incomplete spontaneous abortion (62.8% vs. 67.6%) [4]. This population differs from patients with postpartum RPOC, so a general fertility advantage should not be assumed. The present survey adds information about physician practice and attitudes but cannot resolve these clinical questions.

Bleeding risk is relevant to respondents' safety concerns. In a prospective study of 51 women with RPOC, Sonehara et al. observed massive hemorrhage during follow‐up in 16 of 33 women with power Doppler color scores of 3 or 4 but none of 18 patients with scores of 1 or 2 [8].

Only one of the 22 respondents who performed hysteroscopic surgery reported performing office hysteroscopic surgery. Incognito et al. evaluated office hysteroscopy in a retrospective series of 468 patients with RPOC. The study included hemodynamically stable patients without signs of infection and excluded those with RPOC thickness greater than 30 mm or marked vascularity. Removal was considered complete in 225 of the 333 women who underwent the procedure (67.6%) [9]. These findings suggest that training in office hysteroscopy should address patient selection and the possibility of incomplete removal.

Expectant management should also be addressed in training. In a randomized study following first‐trimester medical termination of pregnancy, misoprostol did not provide a meaningful advantage over expectant management in avoiding subsequent surgery for persistent RPOC [10]. However, these results concerned a different clinical population and may not apply to postpartum RPOC. Similarly, the finding that no respondent selected immediate aspiration or curettage as a basic policy does not establish that these procedures were never used or were inappropriate in individual cases.

Half of the respondents who performed hysteroscopic surgery reported additional treatment, and embolization was common among those who did not perform it. Because the survey did not capture treatment sequence, indications, lesion vascularity, or clinical urgency, the reasons for these interventions could not be determined. Bleeding and residual tissue were reported as separate outcomes.

Interest in training suggests opportunities for collaboration between perinatal clinicians and gynecologic endoscopists. Training could cover interpretation of the clinical course, Doppler findings, diagnostic cavity assessment, and supervised operative techniques appropriate for local resources. Courses should also address referral criteria and preparation for hemorrhage.

Most respondents reported full‐time anesthesiology coverage (68/77, 88.3%), whereas fewer than half reported that interventional radiology was always available (37/77, 48.1%) (Table 1). Educational programs should account for these differences in available resources.

Only 35.7% of respondents who did not use diagnostic hysteroscopy expressed interest in using it, whereas 72.7% of all respondents expressed interest in training. These questions had different denominators and did not assess changes in willingness. Training may also interest physicians who expect to refer patients rather than perform hysteroscopy themselves; however, this possibility was not evaluated in the present survey. Courses can address referral decisions alongside procedural skills. Future educational programs should also evaluate their effects on clinical decision‐making and referral practices.

The findings had several limitations. Recruitment through a society website produced a voluntary sample, although the number of eligible members was unknown, preventing calculation of the response rate or assessment of nonresponse bias. Respondents were predominantly experienced specialists, and many worked in perinatal centers; the findings cannot estimate national uptake among all Japanese obstetrician–gynecologists. More than one respondent may have worked at the same institution; therefore, workplace responses cannot be interpreted as counts of distinct facilities.

Self‐reporting is susceptible to recall bias and differences in interpretation, particularly for questions covering several years. Diagnostic criteria, disease severity, gestational context, treatment timing, and procedure volume were not standardized. The survey's entry question concerned RPOC after delivery, but later questions did not distinguish postpartum from postabortion management.

Analyses were descriptive, and associations between respondent characteristics, workplace resources, and hysteroscopy use were not examined. Twelve respondents reported both diagnostic and operative hysteroscopy use. Finally, the absence of reported diagnostic complications among the 20 users cannot establish safety, and reported operative complications cannot establish incidence without procedural denominators.

Future studies should use de‐identified respondent‐level data to examine associations between hysteroscopy use, clinical experience, and workplace resources. Recruitment and completion should be documented, with diagnostic and operative hysteroscopy use recorded separately. A prospective patient registry could provide procedural denominators and follow‐up data. It should record treatment indications, lesion vascularity, treatment sequence, completeness of removal, hemorrhage, adhesions, and reproductive outcomes.

## 5 Conclusions

Among the 77 responding members of the Japan Society of Perinatal and Neonatal Medicine, a minority used hysteroscopy for RPOC, although overall many expressed an interest in training. Reported concerns included safety, usefulness, and equipment or environment limitations. These findings identify opportunities for targeted education and coordinated access to appropriate expertise. Training could help obstetricians involved in RPOC care in Japan acquire relevant knowledge and practical hysteroscopy skills.

## Author Contributions

**Keita Yatsuki:** conceptualization, methodology, investigation, validation, writing – original draft, formal analysis, data curation, software. **Kazunori Nagasaka:** conceptualization, methodology, investigation, writing – review and editing, writing – original draft, formal analysis, data curation. **Kensuke Sakai:** validation, supervision, visualization, project administration, writing – review and editing. **Akiko Takashima:** writing – review and editing, validation, visualization, project administration. **Kenbun Sone:** conceptualization, validation, writing – review and editing, project administration. **Akemi Koshiba:** validation, visualization, project administration, writing – review and editing. **Osamu Hiraike:** conceptualization, validation, visualization, resources, writing – review and editing, project administration. **Juichiro Saito:** conceptualization, methodology, validation, writing – review and editing, project administration. **Wataru Yamagami:** conceptualization, validation, methodology, visualization, project administration, resources, writing – review and editing.

## Conflicts of Interest

The authors declare no conflicts of interest.

## Supporting information


**Data S1:** De‐identified respondent‐level survey data. The dataset contains all recorded survey responses from 77 physicians, provided in the original Japanese and in English translation, together with a data dictionary and explanatory notes. Respondent identifiers were newly assigned for this supplementary dataset.

**Table S1:** Other reported treatments and proposed indications for hysteroscopy. ^†^Multiple selections were permitted. Values are respondent counts and percentages of the stated questionnaire branch, not patient outcomes or recommended indications. The wording has been translated from the source survey slides. The unanswered operative‐indication item remains in the denominator of 77. RPOC, retained products of conception.

## Data Availability

The de‐identified respondent‐level survey data are provided in Data S1.
