# Supplementary material for: Hysteroscopy for Retained Products of Conception in Japan: A Survey of Clinical Practice and Educational Needs
Source: J Obstet Gynaecol Res. 2026 Nov 8;52(11):e70564. doi: 10.1111/jog.70564 (PMC13745083; doi:10.1111/jog.70564)
Supplement: Supplementary file 1 [file JOG-52-0-s001.docx]

Table S1 Other reported treatments and proposed indications for hysteroscopy

| **Characteristic or response** | **n** | **%** |
| --- | --- | --- |
| **Treatments reported by operative nonusers (N = 53)†** | | |
| Endometrial curettage | 8 | 15.1 |
| Uterine artery embolization | 27 | 50.9 |
| Gonadotropin-releasing hormone antagonist | 4 | 7.5 |
| Hormonal preparations including estrogen–progestin | 10 | 18.9 |
| Hysterectomy | 1 | 1.9 |
| Observation without treatment | 16 | 30.2 |
| Referral | 6 | 11.3 |
| Kampo medicine | 2 | 3.8 |
| Uterotonic medication | 1 | 1.9 |
| Treatment varied by case | 1 | 1.9 |
| No response | 1 | 1.9 |
| **Proposed indications for office hysteroscopy (N = 77)†** | | |
| Worsening imaging findings | 7 | 9.1 |
| Unchanged imaging findings | 18 | 23.4 |
| Improving imaging findings | 12 | 15.6 |
| Disappearance of imaging findings | 13 | 16.9 |
| Symptomatic cases | 9 | 11.7 |
| Asymptomatic cases | 23 | 29.9 |
| All cases | 2 | 2.6 |
| Desire for another pregnancy soon | 30 | 39.0 |
| Unsure | 17 | 22.1 |
| Cases difficult to treat | 1 | 1.3 |
| Unclear imaging or adjunctive diagnosis | 2 | 2.6 |
| Unnecessary | 1 | 1.3 |
| Unable to perform | 1 | 1.3 |
| Inpatient assessment preferred | 2 | 2.6 |
| **Proposed indications for operative hysteroscopy (N = 77)†** | | |
| Worsening imaging such as enlargement or increased flow | 18 | 23.4 |
| Unchanged imaging findings | 29 | 37.7 |
| Improving imaging such as shrinkage or reduced flow | 12 | 15.6 |
| Symptoms such as bleeding | 26 | 33.8 |
| All cases regardless of findings or symptoms | 1 | 1.3 |
| Desire for another pregnancy soon | 35 | 45.5 |
| Unsure | 16 | 20.8 |
| No response | 1 | 1.3 |

Multiple selections were permitted. Values are respondent counts and percentages of the stated questionnaire branch, and not patient outcomes or recommended indications. The wording was translated from the source survey slides. The unanswered operative-indication item remains in the denominator (N = 77). RPOC, retained products of conception.
